# Steady-state ferumoxytol-enhanced MRI: early observations in benign abdominal organ masses and clinical implications

**DOI:** 10.1007/s00261-021-03271-w

**Published:** 2021-10-24

**Authors:** Puja Shahrouki, Ely R. Felker, Steven S. Raman, Woo Kyoung Jeong, David S. Lu, J. Paul Finn

**Affiliations:** 1 Department of Radiological Sciences, University of California Los Angeles, 757 Westwood Plaza, Los Angeles, CA 90095 USA; 2 Diagnostic Cardiovascular Imaging Laboratory, University of California Los Angeles, Peter V. Ueberroth Building Suite 3371, 10945 Le Conte Ave., Los Angeles, CA 90095 USA; 3 Department of Radiology and Imaging Sciences, Samsung Medical Center, Sungkyunkwan University School of Medicine, 81 Irwon-Ro Gangnam-gu, Seoul, 06351 Republic of Korea

**Keywords:** Ferumoxytol, Ultra-small super paramagnetic iron oxide, Contrast media, Steady-state, Magnetic resonance imaging, Renal insuffiency

## Abstract

**Introduction:**

The off-label use of ferumoxytol as a vascular MR imaging agent is growing rapidly. However, the properties of ferumoxytol suggest that it may play an important role in the detection and characterization of abdominal mass lesions.

**Methods:**

Thirty-six patients with benign abdominal mass lesions who underwent MR angiography with ferumoxytol also had T2-weighted HASTE imaging and fat-suppressed 3D T1-weighted imaging. The T1 and T2 enhancement characteristics of the lesions were analyzed and correlated with other imaging modalities and/or surgical findings and/or clinical follow-up.

**Results:**

In all patients with benign masses in the liver (*n* = 22 patients), spleen (*n* = 6 patients), kidneys (*n* = 33 patients), adrenal (*n* = 2 patients) and pancreas (*n* = 4 patients), based on the enhancement characteristics with ferumoxytol, readers were confident of the benign nature of the lesions and their conclusions were consistent with correlative imaging, tissue sampling and follow-up. One patient with a suspicious enhancing 2F Bosniak renal cyst had renal cell carcinoma confirmed on biopsy.

**Conclusion:**

Ferumoxytol-enhanced MRI can increase diagnostic confidence for benign abdominal masses and can increase the conspicuity of mass lesions, relative to unenhanced MRI.

**Graphic Abstract:**

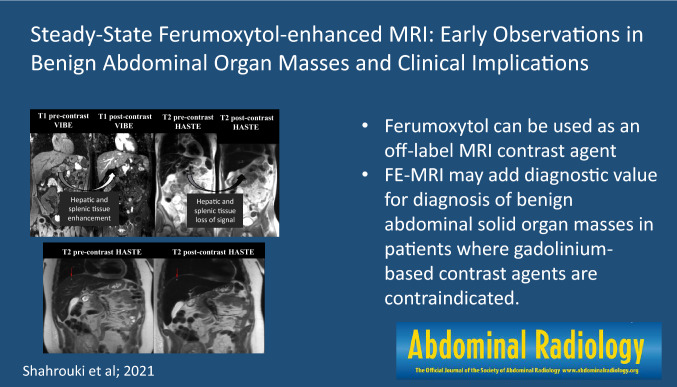

## Introduction

Whereas experience is growing rapidly with the use of ferumoxytol for vascular and cardiac MRI, little has been reported about its potential for imaging of solid organs [1, 2]. This is gaining relevance as the off-label use of ferumoxytol is increasing in patients with renal impairment or complex vascular diseases. The pharmacokinetics and relaxometry of ferumoxytol are very different from all of the available GBCAs, and data are lacking about its behavior in extracranial solid organ lesions. Ferumoxytol was originally developed as an intravascular contrast agent, but was redirected to the iron replacement therapy market and an imaging indication was never pursued. Although ferumoxytol is limited to off-label use for MRI, its mixed T1 and T2 shortening properties make it a versatile and powerful vascular contrast agent. These magnetic properties coupled with a long intravascular half-life (15 h) allow for extended steady-state imaging giving positive (hyperintense) and negative (hypointense) enhancement on T1- and T2-weighted images, respectively [1]. For organs with a high blood volume content, such as liver and spleen, the relative signal changes induced by ferumoxytol should parallel those of the blood vessels.

The limited published experience on the appearance of common benign lesions with FE-MRI may result in uncertainty among radiologists for characterization of incidental lesions. The aim of our study was to describe our early experience of steady-state FE-MRI imaging in a variety of benign masses in the liver, spleen, kidneys, pancreas and adrenal glands (with focus on imaging technique, enhancement characteristics and clinical impact).

## Materials and methods

This is an IRB-approved and HIPAA-compliant study for retrospective review of prospectively collected data. All patients provided written informed consent. Thirty-six adult patients who underwent FE-MRI between October 2013 and January 2018 with retrospectively identified abdominal solid organ body masses on FE-MRI were included in the study. Patients that had lesions highly suspicious for or with confirmed abdominal malignancy were excluded from review. Patients with only simple renal cysts and no other type of focal lesion were not included for review as the majority of patients undergoing FE-MRI have chronic kidney disease with concurrent renal cysts. The primary imaging indications for FE-MRI were vascular.

FE-MRI imaging was performed either on a 3.0 T whole body MR imaging system (Magnetom TIM Trio (*n* = 12), Magnetom Prisma Fit (*n* = 16) or Magnetom Skyra (*n* = 4); Siemens Medical Solutions, Malvern PA) or on a 1.5 T whole body MR imaging system (Magnetom TIM Avanto (*n* = 4), Siemens Medical Solutions, Malvern, PA). Ferumoxytol was administered intravenously as a bolus over 15 s with pre- and postcontrast acquisitions if clinically indicated prior to April 2015, and from April 2015 ferumoxytol was administered intravenously by slow-infusion per FDA recommendations [3]. The imaging sequences included high resolution, 3D T1 contrast-enhanced MR angiography (*n* = 36, 100%), pre- and post-contrast T2 weighted Half-Fourier Acquisition Single-shot Turbo spin Echo (HASTE; *n* = 18, 50%), post-contrast T2 weighted HASTE without pre-contrast HASTE (*n* = 15, 42%) and pre- and post T1 weighted fat suppressed 3D gradient echo (*n* = 8, 22%). The masses were retrospectively identified and correlated with histopathology, if available. If available within 36 months of the FE-MRI, contrast-enhanced or positron emission tomography (PET) imaging closest to the FE-MRI was used for review if the same lesion was present and no interventions to the lesion were carried out in the interim. The patients were also retrospectively reviewed for ultrasonographic and non-contrast cross-sectional imaging of the target lesion up to six months prior to the FE-MRI to identify if any lesions may have been missed or inadequately assessed.

## Results

The study cohort comprised 36 adult patients who underwent FE-MRI (median age 60.0, interquartile range 48.9–74.5 years, 58% male), with 10 different types of solid organ lesions (Table 1). The average baseline creatinine and estimated glomerular filtration rate (eGFR) was 2.78 mg/dL ± 2.13 mg/dL and 30.33 mL/min/1.73 m^2^ ± 21.73 mL/min/1.73 m^2^ respectively. Twenty-one (58%) of 36 patients had non-contrast CTs or ultrasonography studies available within 6 months prior to the FE-MRI. Common for all benign lesions in these 21 patients was incomplete characterization on non-contrast CT and US prior to the FE-MRI. These benign lesions could only confidently be diagnosed as benign when long-term interval imaging was available or when the lesions were > 1 cm.

**Table 1:** Identification of solid organ mass lesions

	Suspected type of lesion	Number of patients studied (n)	Number of patients with comparative contrast-enhanced imaging (%)	Comparative contrast-enhanced imaging radiology report	Number of patients with biopsy or autopsy proven lesion (%)	Biopsy report
Liver	Cyst^a^	18	6 (28)	PET-CT (*n* = 2), contrast-enhanced CT (*n* = 4): cyst	0 (0)	…
	Biloma	1	1 (100)	GE-MR: nothing visualized^b^	1 (100)	Biloma
	Hemangioma	4	2 (50)	GE-MR (*n* = 1) and contrast-enhanced CT (*n* = 1): hemangioma	0 (0)	…
	Indeterminate	1	1 (100)	GE-MR: “FNH-like” lesions of congenital heart disease	0 (0)	…
Kidney	Complex renal cyst	2	1 (50)	GE-MR: complex renal cyst	1 (50)	Renal cell carcinoma
	Simple renal cyst^a^	30	7 (23)	CE-CT (*n* = 3): indeterminate; CE-CT (*n* = 1), GE-MR (*n* = 2), PET-CT (*n* = 1): cyst	0 (0)	…
	Angiomyolipoma	1	0 (0)	…	0 (0)	…
Pancreas	Cyst^a^	4	1 (25)	CE-CT (*n* = 1): cyst	0 (0)	…
Spleen	Cyst^a^	6	2 (33)	CE-CT (*n* = 1): indeterminate; CE-CT (*n* = 1): cyst	1 (0)	Cyst
Adrenal	Adenoma	2	0 (0)	…	0 (0)	…

^a^Includes patients with polycystic disease, hemorrhagic cysts and infectious cysts

^b^Not visualized on retrospective review and therefore considered novel lesion

Five (14%) patients had an abdominal mass suspicious for malignancy (3 renal, 1 hepatic and 1 splenic lesion) who underwent FE-MRI. All five (100%) patients had undergone a contrast-enhanced MR study after either non-contrast CT (*n* = 2) or duplex ultrasound (*n* = 3) were indeterminate. Malignancy was confidently excluded in four (80%) of five patients on FE-MRI without the need for additional imaging. Biopsy was carried out in one of the four patients where FE-MRI imaging had confidently excluded malignancy, where histopathological results confirmed a benign splenic cyst. None of the other three lesions have been found to be malignant to date (range of follow up 21–51 months). In the patient where an abdominal mass could not be excluded on FE-MRI, a type 2F Bosniak renal cyst was described and histopathological correlation after one year demonstrated a confined cystic renal cell carcinoma (RCC). The patient had no evidence of recurrent or metastatic disease to date (two years post-resection).

### Liver

**Fig. 1:**
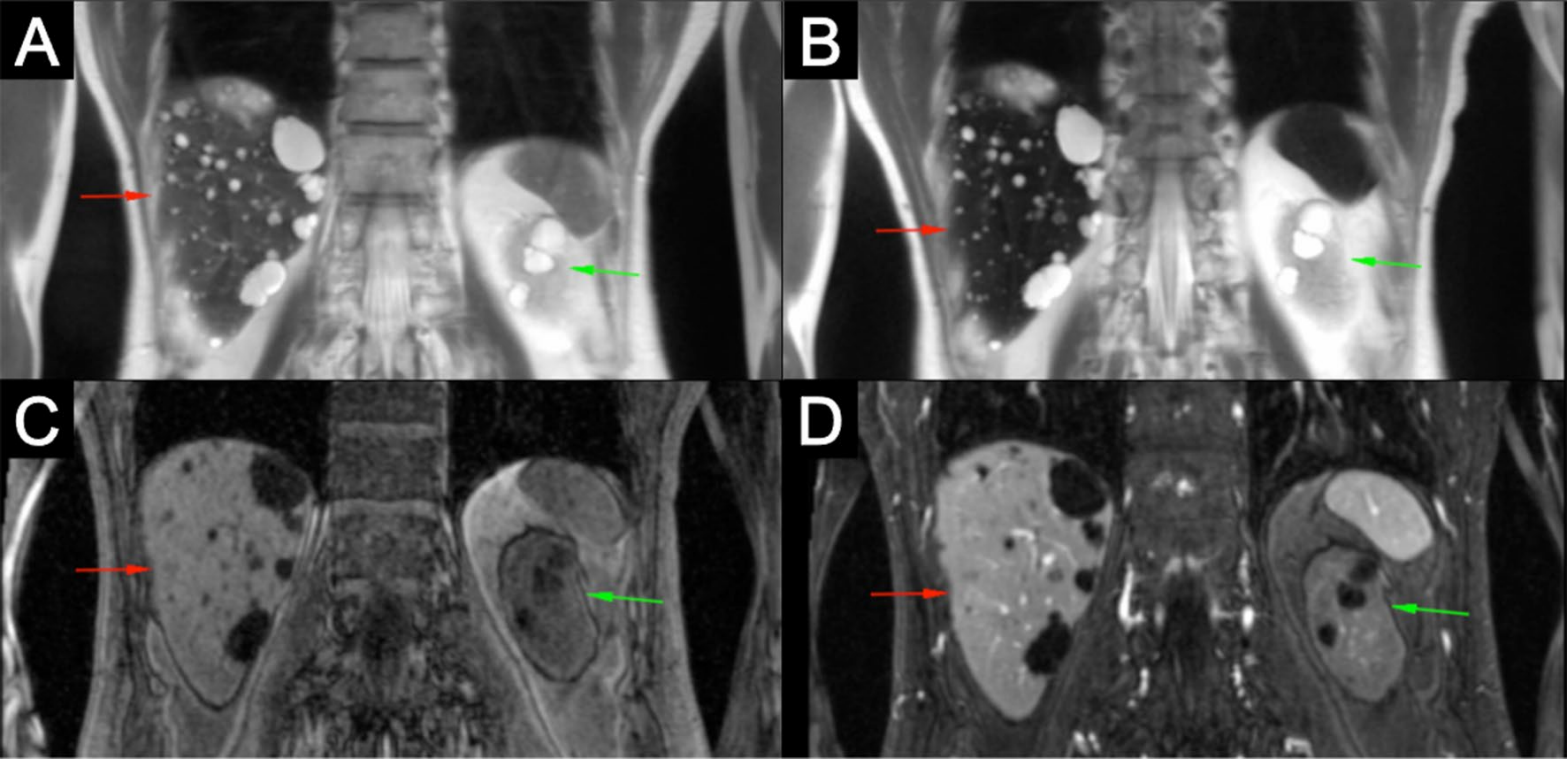
3.0 T ferumoxytol-enhanced MRI of 63-year-old male with polycystic liver and kidneys. Pre-contrast T2 (**a**) and T1 (**c**) images show typical cystic appearance with hyperintensity and hypointensity, respectively. Post-contrast images (**b** and **d**) reveal increased conspicuity of cysts with administration of ferumoxytol for both hepatic (red arrows) and renal cysts (green arrows), but pronounced improvement for hepatic cysts on T2 images and renal cysts on T1 images

**Fig. 2:**
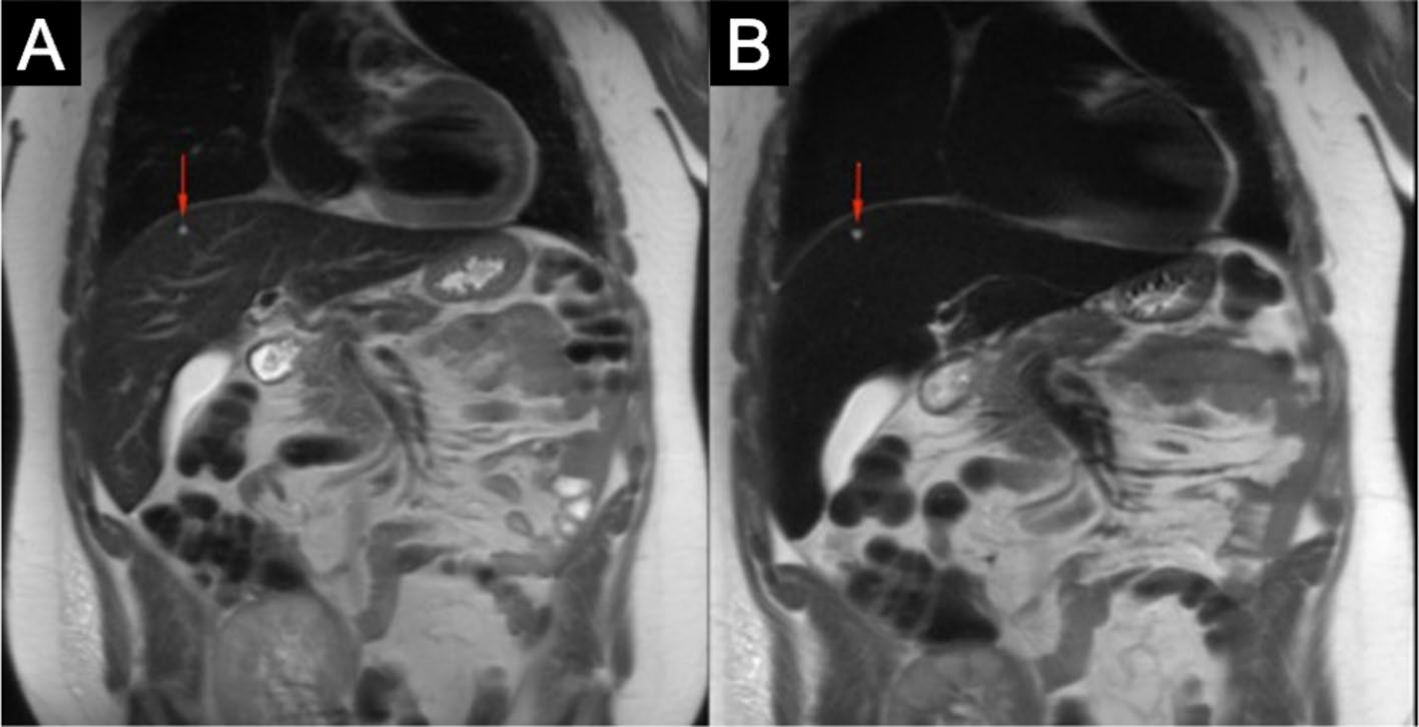
3.0 T ferumoxytol-enhanced MRI in a 20 year-old male with a single hepatic cyst. Pre- (**a**) and post-contrast (**b**) T2 acquisitions show increased conspicuity of a 4 mm hyperintense benign cyst (red arrow) as the normal hepatic tissue's signal intensity is suppressed

**Fig. 3:**
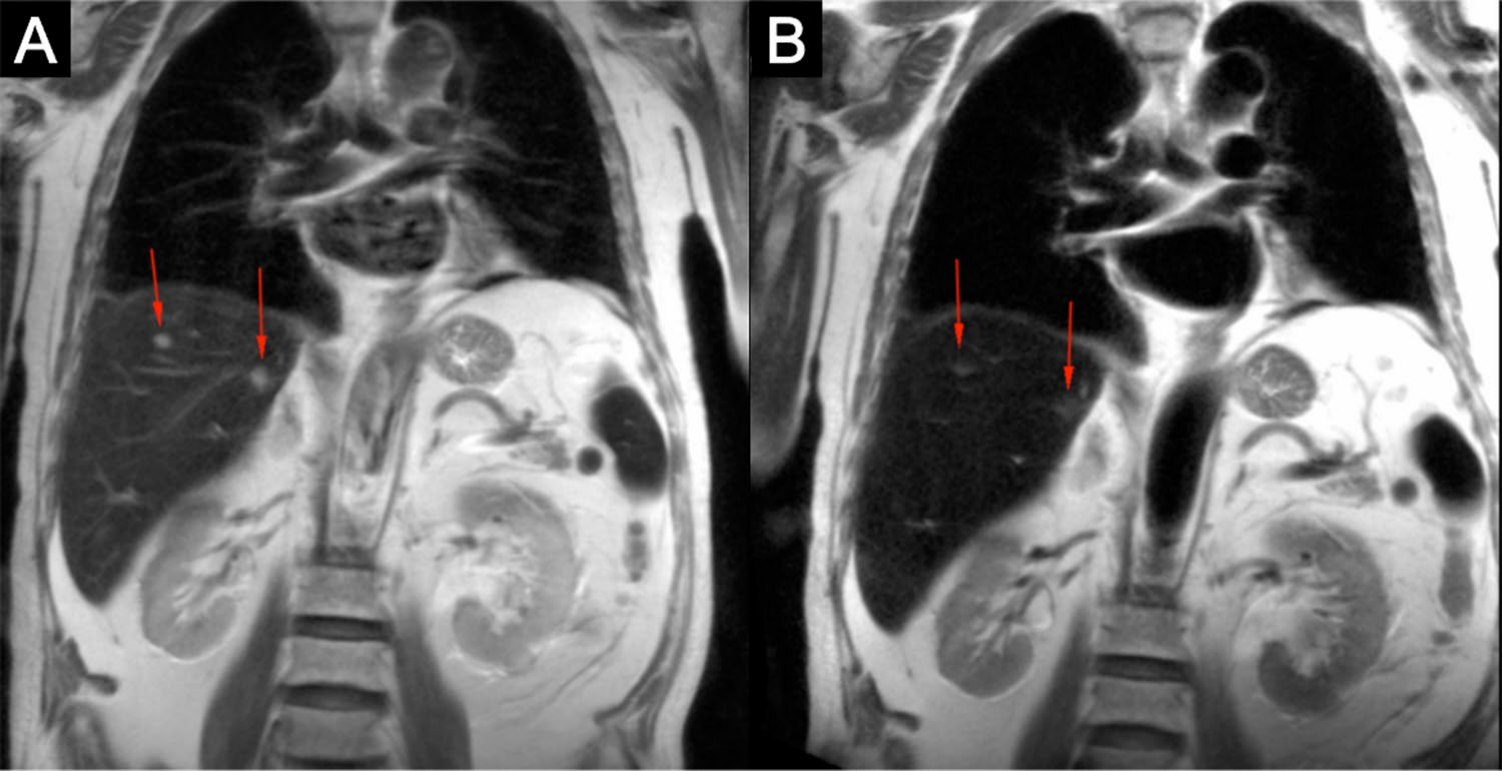
3.0 T ferumoxytol-enhanced MRI in a 74 year-old male with hemangiomas. Pre-contrast (**a**) T2 HASTE acquisitions show round hyperintense lesions (red arrows) indistinguishable from cysts without contrast. Post-contrast (**b**) T2 HASTE acquisitions show loss of signal for the lesions (red arrows) which is suggestive of hemangiomas

There were four different types of benign solid liver lesions identified in 24 patients (Table 1). The most common benign lesion was a hepatic cyst which has a typical appearance on MRI of hypo- and hyperintensity on T1- and T2-weighted images respectively [4, 5]. With the addition of ferumoxytol, the conspicuity of cysts increases as the signal intensity of the normal liver tissue decreases on T2 because of the relatively high blood pool fraction in the liver (Fig. 1). The remarkable signal drop of the liver on post-contrast T2 HASTE is especially helpful in confidently identifying very small cysts as the conspicuity may increase dramatically with the administration of ferumoxytol (Fig. 2). Also, on T1-weighted images, normal hepatic parenchyma enhances uniformly because of the large blood volume in the sinusoids and this generally serves to highlight the majority of hypointense masses which are less vascular (with the notable exception of hemangioma). The unique imaging features of the liver seen with ferumoxytol were established for other superparamagnetic iron oxides (SPIOs) more than two decades ago [6, 7]. The utility of contrast-enhancement with ferumoxytol is probably best exemplified by small lesions (≤ 10 mm) that would otherwise not have been characterized on non-contrast CT or MR studies and could potentially have led to unwarranted follow-up imaging or interventions when malignancy could not be confidently excluded. Similarly, contrast-enhancement with ferumoxytol allows for the confident diagnosis of other benign lesions such as hepatic hemangiomas (Fig. 3).

One common focal hepatic lesion that is at risk of being underdiagnosed with FE-MRI is focal nodular hyperplasia (FNH), as might be suggested by the lack of such lesions found in our patient cohort. As these lesions typically enhance during the arterial phase and are difficult to discern from normal hepatic tissue in the later post-contrast phases, FNHs are probably missed on steady-state imaging as previous studies on gadofosveset trisodium have established [8]. Oftentimes, FNH is important to distinguish from less common lesions such as hepatic adenomas, hepatocellular carcinoma and metastases [9], and therefore, future studies should confirm that characterization of FNH is limited on steady-state FE-MRI.

Another potential area of investigation for FE-MRI in liver imaging is diffuse disease, such as fibrosis or cirrhosis; diseased liver tissue is less elastic and associated with portal hypertension where there is restricted portal blood flow, possibly leading to less enhancement with ferumoxytol. Indeed, it is well-established that portal venous flow decreases in cirrhotic livers, but there is a hepatic arterial buffer response that compensates so that the total hepatic blood flow remains constant [10, 11]. It is however unclear when exactly this response is activated.

### Spleen

**Fig. 4:**
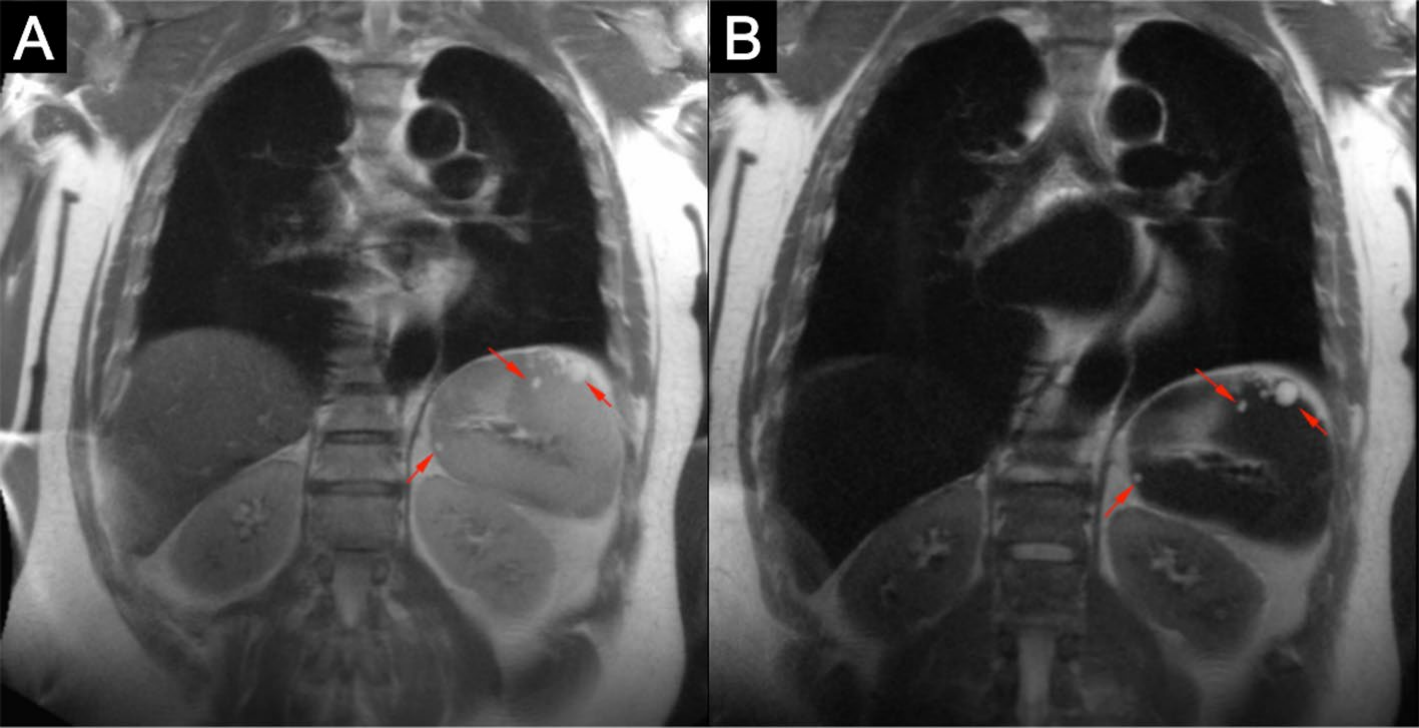
3.0 T ferumoxytol-enhanced MRI in a 60-year-old male with multiple splenic cysts. Pre- (**a**) and post-contrast (**b**) T2 acquisitions show increased conspicuity multiple benign cysts (red arrows) so that even subcentimeter cysts can be distinguished from the normal splenic tissue that loses signal with the addition of ferumoxytol

**Fig. 5:**
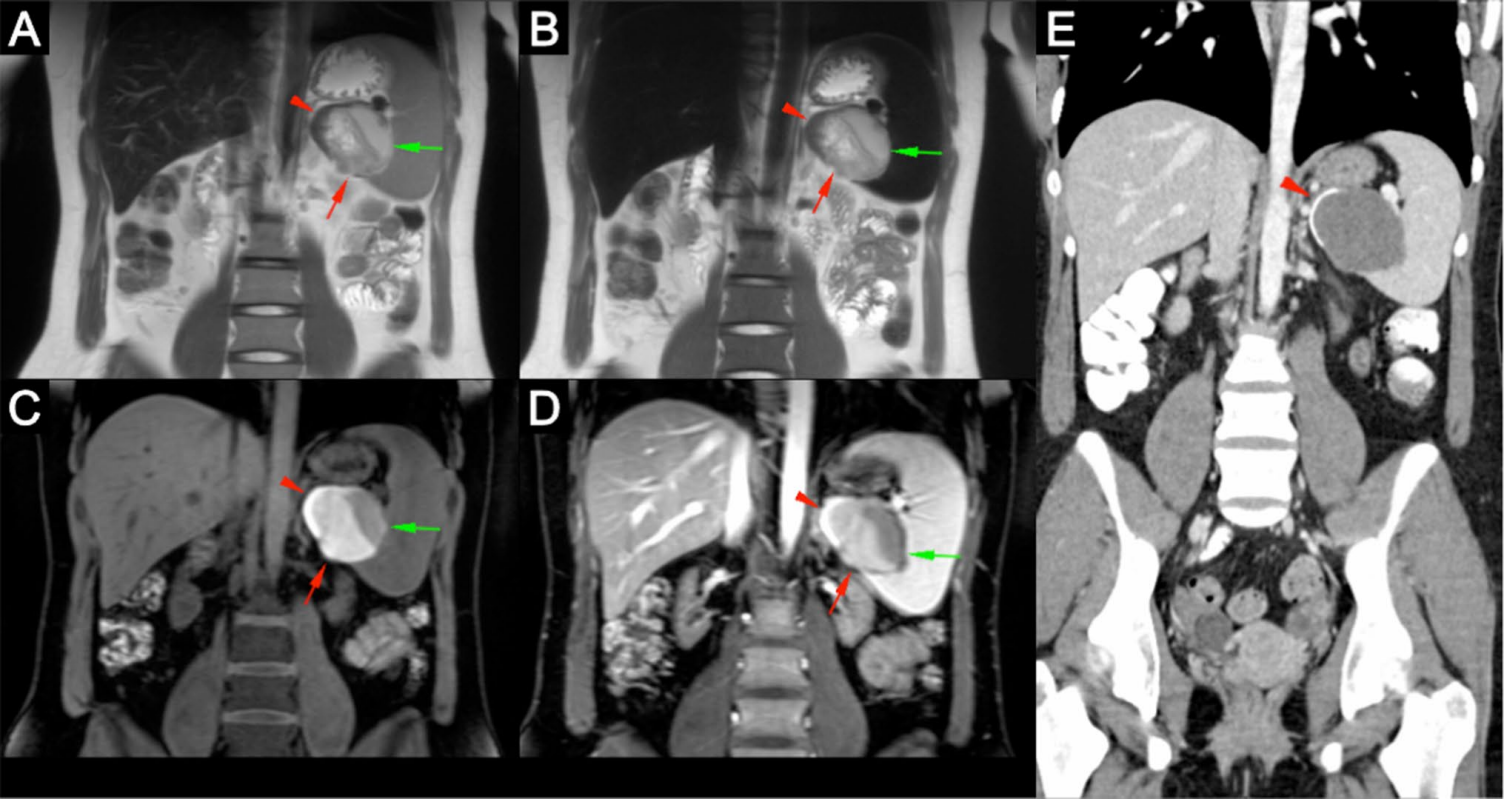
20 year-old female with an indeterminate splenic mass on ultrasound and contrast-enhanced CT suspicious for malignancy. Pre- and post-contrast T2 (**a** and **b**) and T1 (**c** and **d**) 3.0 T ferumoxytol-enhanced MRI acquisitions show a large splenic lesion with a cystic component (green arrows), and solid component (red arrows) with heterogenous T2 intensity and T1 hyperintensity without contrast enhancement suggestive of proteinaceous content. The solid component also has a linear rim (red arrowhead) of T2 hypointensity and T1 hyperintensity suggestive of a calcification. The prior contrast-enhanced CT study (**e**) confirms absence of contrast enhancement in the entirety of the lesion and a hyperdense rim (red arrowhead) suggestive of calcification. Altogether the constellation of findings was suggestive of a hemorrhagic cyst which was later confirmed on biopsy

Six patients were identified to have benign cystic lesions in the spleen (Table 1). Even more than the liver, normal spleen undergoes signal loss on T2 imaging with ferumoxytol, owing to its large blood volume. This mechanism increases the conspicuity of small lesions so that confident diagnosis of small lesions can be made. As benign lesions in the spleen are not as common as in the liver, the confident differentiation of these lesions from malignant lesions becomes increasingly important [12, 13]. Simple cysts do not enhance with ferumoxytol and are particularly conspicuous on post-contrast T2 images when they are hyperintense in a hypointense background (Fig. 4). In addition, the absence of contrast-enhancement with ferumoxytol can provide crucial differentiation of intrinsically T1 hyperintense cysts (containing blood products or proteinaceous material) from malignant lesions (Fig. 5).

### Kidney

**Fig. 6:**
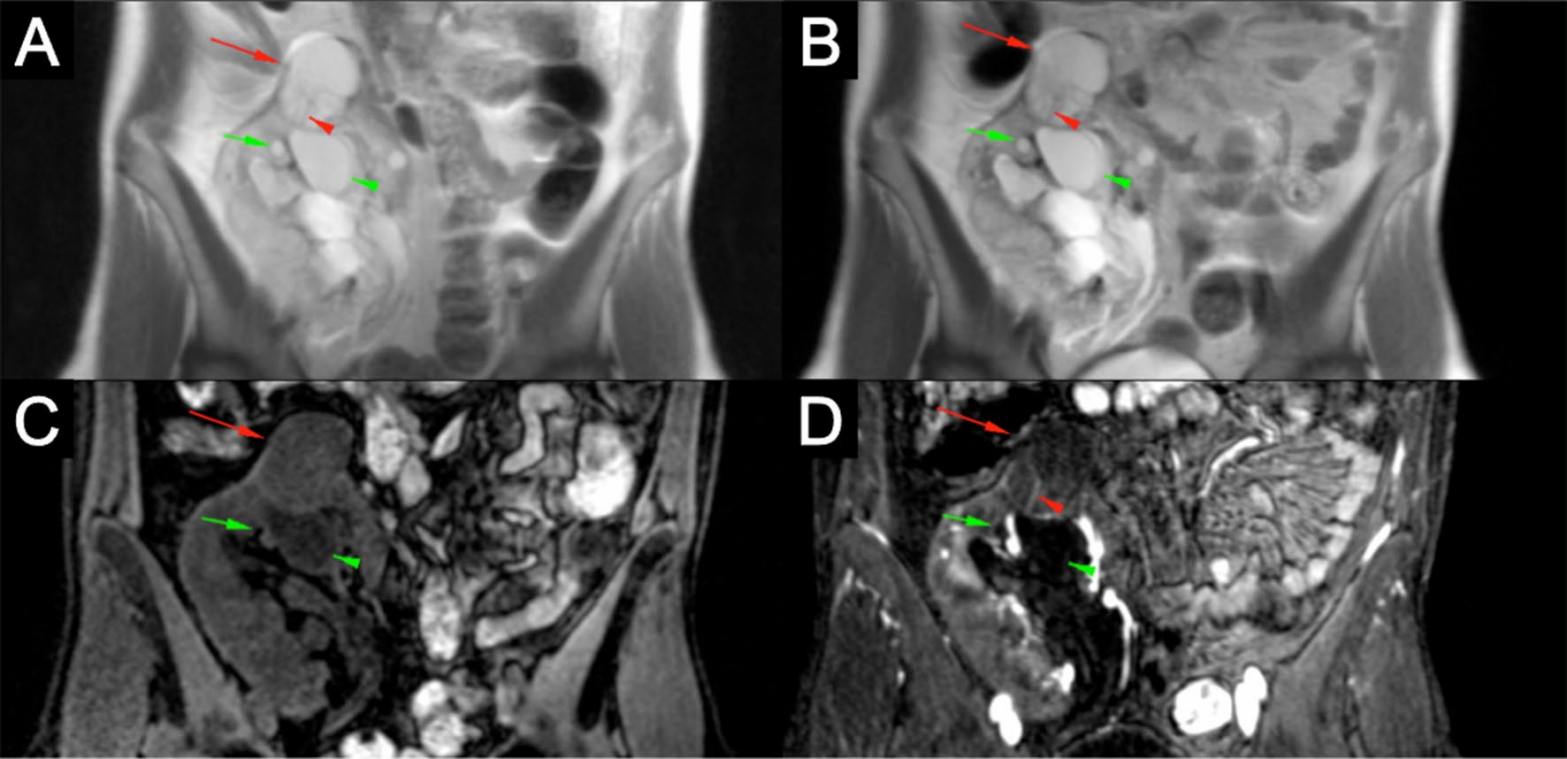
3.0 T ferumoxytol-enhanced MRI in a 30-year-old female with an indeterminate renal mass in the superior pole of a right renal allograft on ultrasound. Pre- and post-contrast T2 (**a** and **b**) and T1 (**c** and **d**) images show a septated cyst in the superior pole of the kidney (red arrows) with perceived but non-quantifiable enhancement (red arrowheads) and lack of solid enhancing component consistent with a 2F Bosniak renal cyst. Additionally, multiple simple benign cysts are seen that are T2 hyperintense and T1 hypointense with no contrast-enhancement (simple cysts, green arrows) and T2 hyperintense and intrinsically T1 hyperintense with no contrast enhancement (hemorrhagic cysts, green arrowheads). Histopathologic analysis of the 2F Bosniak renal cyst after 1 year demonstrated a confined cystic renal cell carcinoma

In the kidneys, three different types of solid renal lesions were identified in 33 patients (Table 1). Simple benign renal cysts are, such as hepatic and splenic cysts, oval-shaped with homogeneous T1 hypointensity and T2 hyperintensity with no contrast enhancement (Figs. 1 and 6). Sometimes benign renal cysts may hemorrhage either upon trauma or spontaneously, and in these cases the cysts can have increased signal intensity because of proteinaceous or hemorrhagic content, albeit with continued negative enhancement [14, 15] (Fig. 6). The complexity of cystic renal masses can be evaluated according to the Bosniak Classification System, which was originally designed to classify cysts based on CT findings, later found to be compatible with MRI [16, 17] (Table 2). Cysts are more prevalent in patients with renal impairment, especially those on long-term dialysis [18], which is the main patient population undergoing FE-MRI at our institution. Therefore, confident assessment of the complexity of cysts is important for early detection of cancers, and determination of contrast-enhancement is a crucial step in the classification of renal cysts. As Fig. 6 shows, contrast enhancement of cysts with ferumoxytol can be used to classify the complexity of cysts. A previous study on the utility of FE-MRI for assessment of kidney transplant recipients also found that contrast-enhancement of renal cysts could determine complexity in two cases that were both found to be RCC [19]. As the overall life expectancy of patients with ESRD is increasing [20], the value of early detection of RCCs becomes more important and an area of investigation should be FE-MRI screening of high-risk patients with ESRD [21].

**Table 2:** The Bosniak classification of renal cysts. Adapted from Refs. [16, 17]

Bosniak classification	Criteria	Management
I	Simple cyst with hairline-thin wall with no septa, calcifications, solid components or enhancement	No follow-up needed
II	Cyst which may contain: few hairline-thin septa without measurable enhancement, fine calcification or a short segment of slightly thickened calcification in the wall or septa, uniformly high-attenuating lesions (< 3 cm) that are sharply marginated and do not enhance	No follow-up needed
IIF	Cysts which may contain: multiple hairline-thin septa, immeasurable enhancement of septum or wall, minimal thickening of wall or septa, calcification of wall or septa, no enhancing soft-tissue components, generally well-marginated. Also includes uniformly high-attenuating lesions (> 3 cm)	Imaging follow-up to assess for malignancy
III	Cyst with thickened irregular or smooth walls or septa and in which measurable enhancement is present, no distinct enhancing soft tissue enhancement. Also includes complicated hemorrhagic or infected cysts, multilouclar cystic nephroma and cystic neoplasms	Surgical excision with histological examination to assess for malignancy
IV	Cyst with thickened irregular or smooth walls or septa and in which measurable enhancement is present with distinct soft-tissue enhancement independent of wall or septa	Surgical excision with histological examination to assess for malignancy

### Adrenal glands

**Fig. 7:**
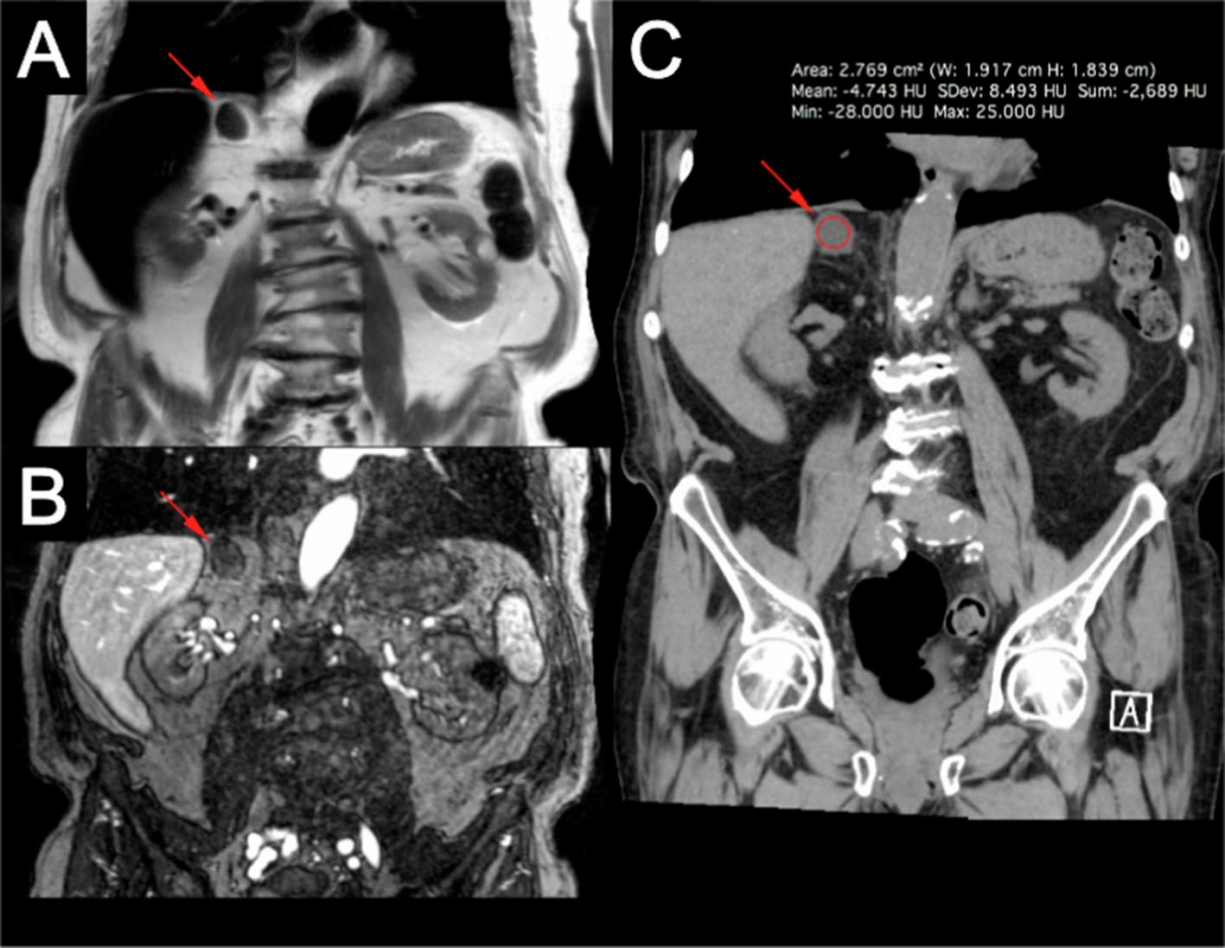
89-year-old male with a known adrenal adenoma. Post-contrast T2 (**a**) and T1 (**b**) 3.0 T ferumoxytol-enhanced MRI acquisitions show a solid enhancing adrenal lesion (red arrows). Follow-up non-contrast CT (C) confirms that the lesion is a lipid-rich adrenal adenoma (red arrow, red circle)

In the adrenal glands, two adenomas were identified in two patients (Table 1). Incidental lesions other than adenomas are uncommon in the adrenal glands and most adrenal adenomas that have high intracellular lipids can be confidently diagnosed with non-contrast CTs, but differentiation from metastasis remains an important task for the radiologist in adenomas with low lipid content [22–24]. Dynamic contrast-enhancement patterns and chemical shift MRI images can be used for the differential diagnosis [24], and it is still unclear if steady-state imaging with ferumoxytol can be used to confidently distinguish an adenoma from a metastasis. From what can be seen from our limited cases, adrenal adenomas show uniform contrast enhancement which is especially conspicuous on T2 HASTE images as seen in Fig. 7. Interestingly, the adrenal gland is the abdominal organ with the most blood flow per unit weight and has a higher blood volume per unit weight than the liver [25], which makes it an organ with significant ferumoxytol-enhancement like the liver, spleen and kidneys. Gunn et al. showed that delayed ferumoxytol-enhanced studies with images taken 48 h after contrast administration show a heretofore unexplainable drop in signal on T2 imaging (positive enhancement) [26], and further studies should be carried out to determine the contrast dynamics of the adrenal glands.

### Pancreas

**Fig. 8:**
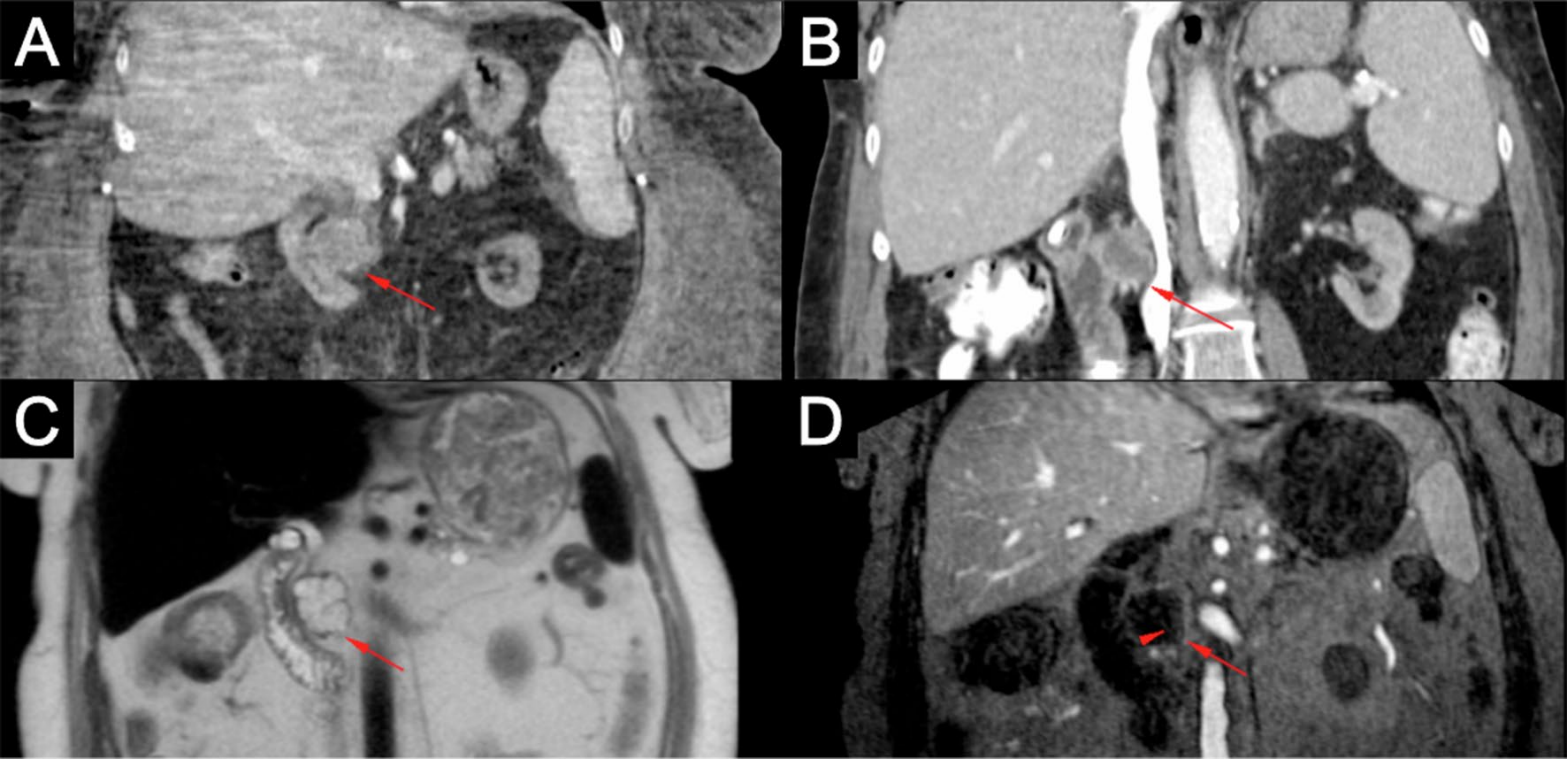
66 year-old female with a known stable cystic lesion in the pancreatic head. Contrast-enhanced CT 1 year prior to (**a**) and 2 years after (**b**) 1.5 T ferumoxytol-enhanced MRI (**c**, **d**) show a large cystic lesion (red arrows). Post-contrast T2 and T1 acquisitions show clustering of multiple cysts in the lesion (red arrows), thin septation (red arrowhead) and lack of suspicious enhancing components suggestive of a serous cystadenoma or intraductal papillary mucinous neoplasm

Four patients were identified with benign cystic pancreatic lesions (Table 1). All lesions were determined to be cystic and none of the patients had a history of acute pancreatitis. It is difficult to differentiate benign cystic pancreatic lesions based on imaging alone and therefore the clinical presentation, age, sex and histological findings are used in conjunction for diagnosis [27, 28]. Figure 8 shows a large cystic lesion within the pancreatic head that is better characterized on MRI than CT, although the exact classification cannot be determined with the imaging features (multi-locular and located in pancreatic head) and history (asymptomatic elderly female) alone. The differentiation of these benign pancreatic cystic lesions is important as they have different malignant potential [27, 28]. Although it remains unclear how benign cystic pancreatic lesions can be differentiated with FE-MRI, delayed ferumoxytol-enhanced MRI studies of pancreatic adenocarcinomas suggest that ferumoxytol can aid in the delineation of pancreatic tumors [29]. Therefore, further imaging studies with pathological correlation are required to determine how ferumoxytol-enhancement may help in classifying focal pancreatic lesions, especially differentiating between benign and malignant lesions.

### Safety of Contrast Agents

The safety profile of contrast media is an important factor to consider when deciding on the appropriate agent for patients with renal impairment, an area which has caused much confusion in clinical practice. Historically, one of the primary concerns with GBCAs has been the perceived risk of nephrogenic systemic fibrosis (NSF), an increasingly rare complication, and recent studies have raised concerns about gadolinium deposition in brain and bone, primarily associated with linear GBCAs [30, 31]. Although the clinical significance of gadolinium tissue deposition has not been established, the potential for unknown long-term effects causes unease in clinical practice [32]. However, there is no known deposition of GBCAs within the abdominal organs that would impede with future diagnostic assessments. In contrast, if ferumoxytol has previously been administered, subsequent MRI examinations of the liver can be confounded and potentially mask malignancy, as was described in a case report by McCullough et al. [33]. Ferumoxytol can result in a change of T2 MRI characteristics of the liver and spleen for months, due to iron storage within the organs [34, 35]. Although liver storage of ferumoxytol is a normal and physiological response, this caveat should be known to radiologists interpreting images in patients who have received ferumoxytol for diagnostic, or therapeutic purposes.

Another factor to consider when deciding on the appropriate contrast agent is rate of adverse events. For ferumoxytol, rare but serious hypersensitivity reactions were reported in the post-marketing surveillance period, attributed to fast bolus administration at therapeutic doses [3, 36]. However, meta-analysis of multiple single-center studies and a multi-center Registry study found no serious adverse events with the diagnostic use of ferumoxytol when administered as a slow infusion, while commercially available GBCAs also have a very low severe allergic-like adverse events rate of 0.52 per 10 000 injections [37–39]. Consistent with these data, there were no adverse events in our study.

## Conclusion

Early results of FE-MR in solid organs show that ferumoxytol is a useful tool in characterizing benign abdominal masses. By analyzing the enhancement characteristics on T1-weighted and T2-weighted images and comparing these to histological and other radiological studies when available, it was possible to characterize benign lesions successfully. As expected, ferumoxytol shares many of the enhancement patterns of gadolinium-based contrast agents for common benign lesions. A unique property of ferumoxytol as a contrast agent is the increased conspicuity of lesions in hypervascular organs such as the liver, kidney and spleen as lesions enhances less than surrounding normal tissue because of ferumoxytol’s high T2 relaxitivity. However, a limitation with our study is that many of the lesions we describe, e.g. lipid-rich adrenal adenomas, could be confidently characterized as benign on non-contrast studies, and in such cases FE-MRI may provide little additional value. We suggest that the highest benefit of contrast-enhancement of benign lesions with ferumoxytol can be found in small lesions that may either be missed or incorrectly characterized on imaging studies without contrast. Our findings suggest that ferumoxytol may add diagnostic value for diagnosis of benign solid organ mass lesions in patients where GBCA are contraindicated. Further studies need to be carried out to determine the diagnostic efficacy of FE-MRI in malignant abdominal lesions.

## Data Availability

The datasets used and/or analysed during the current study are available from the corresponding author on reasonable request.
